# Elastin is responsible for the rigidity of the ligament under shear and rotational stress: a mathematical simulation study

**DOI:** 10.1186/s13018-023-03794-6

**Published:** 2023-04-19

**Authors:** Yuki Naya, Hiroki Takanari

**Affiliations:** 1 Division of Interdisciplinary Research for Medicine and Photonics, Institute of Post-LED Photonics, Tokushima University, 3-18-15, Kuramoto, Tokushima, 770-8503 Japan; 2 Graduate School of Medical Sciences, Tokushima University, Tokushima, Japan

**Keywords:** Elastin, Collagen, Ligament, Simulation, Stress, Mechanical response

## Abstract

**Background:**

An accurate understanding of the mechanical response of ligaments is important for preventing their damage and rupture. To date, ligament mechanical responses are being primarily evaluated using simulations. However, many mathematical simulations construct models of uniform fibre bundles or sheets using merely collagen fibres and ignore the mechanical properties of other components such as elastin and crosslinkers. Here, we evaluated the effect of elastin-specific mechanical properties and content on the mechanical response of ligaments to stress using a simple mathematical model.

**Methods:**

Based on multiphoton microscopic images of porcine knee collateral ligaments, we constructed a simple mathematical simulation model that individually includes the mechanical properties of collagen fibres and elastin (fibre model) and compared with another model that considers the ligament as a single sheet (sheet model). We also evaluated the mechanical response of the fibre model as a function of the elastin content, from 0 to 33.5%. Both ends of the ligament were fixed to a bone, and tensile, shear, and rotational stresses were applied to one of the bones to evaluate the magnitude and distribution of the stress applied to the collagen and elastin at each load.

**Results:**

Uniform stress was applied to the entire ligament in the sheet model, whereas in the fibre model, strong stress was applied at the junction between collagen fibres and elastin. Even in the same fibre model, as the elastin content increased from 0 to 14.4%, the maximum stress and displacement applied to the collagen fibres during shear stress decreased by 65% and 89%, respectively. The slope of the stress–strain relationship at 14.4% elastin was 6.5 times greater under shear stress than that of the model with 0% elastin. A positive correlation was found between the stress required to rotate the bones at both ends of the ligament at the same angle and elastin content.

**Conclusions:**

The fibre model, which includes the mechanical properties of elastin, can provide a more precise evaluation of the stress distribution and mechanical response. Elastin is responsible for ligament rigidity during shear and rotational stress.

**Supplementary Information:**

The online version contains supplementary material available at 10.1186/s13018-023-03794-6.

## Background

The ligament is an important organ that connects one bone to the other to support the joints, maintain posture, and facilitate movement. Mechanical loading beyond the motion range or in a direction different from the motion range of the joint may cause damage or rupture of the ligaments, such as medial collateral ligament injuries in the elbows of baseball players and anterior cruciate ligament injuries in the knees of football players. Recently, the number of ligament injuries and reconstructive surgeries has increased with the diversification and sophistication of different sports and growing sports population [1, 2]. For example, rupture of the anterior cruciate ligament occurs at a rate of 30–78 per 100,000 people [3]. Once ligaments are damaged or ruptured, treatment including reconstructive surgery, transplantation, and platelet-rich plasma injection is performed, all of which require a long time before complete healing and may not completely restore the original function [4]. Therefore, in sports medicine, it is important to thoroughly understand the mechanical properties and responses of the ligament and the mechanisms of injury or rupture to prevent ligament damage. Many mathematical simulations [5], as well as animal studies [6, 7], have been conducted.

Ligaments mainly consist of water, cells, and extracellular matrix (ECM) components, including type-I collagen and elastin, which bind directly or indirectly via proteoglycans, laminin, and other adhesion molecules [8, 9]. Collagen, which accounts for 85–90% of the ECM dry weight, as the major component of ligaments, provides them with moderate rigidity and viscoelasticity [10]. On the other hand, elastin accounts for approximately 10% of the dry weight of the ECM and is considered to render the ligaments extensible [11–13].

To understand the mechanical response of the ligaments, experiments have been conducted to verify the stress–strain relationship and changes in shape by applying mechanical loads to the ligaments obtained from experimental animals. As these animal experiments used a ligament as a single tissue, it was difficult to determine the mechanical properties of each ligament component. Several attempts have been made to determine the mechanical properties of elastin before and after its removal by elastase [12, 13]. However, it remains difficult to examine each of the crosslinking elements such as laminin and proteoglycans.

Based on experimental animal data, mathematical simulations have been conducted extensively to predict the mechanical response of ligaments. Recent improvements in computer performance have led to more precise simulations, such as the generation of models based on accurate anatomical data obtained by computed tomography and other diagnostic imaging methods, and the mechanical response of collagen fibres at the microscopic level has also been actively studied [14]. Computer simulations can also be used to predict parameters for which the exact values are not known by comparing them with actual measurements. One pertaining problem is that many simulation studies consider the ligament to be a uniform collagen fibre bundle [15–17] or sheet [18, 19], ignoring the mechanical properties of its other major components, elastin, and the interaction between collagen and elastin. Although the content and distribution of elastin have been studied at the molecular level and their unique mechanical properties have been tackled, their anatomical or physiological role in ligaments has not been fully understood.

We hypothesised that models that consider the individual properties and distributions of collagen and elastin would show different mechanical responses than models in which ligaments are treated as a single tissue or those that do not include elastin. To this end, we constructed a simple model including collagen and elastin based on two-photon microscopy images of porcine knee collateral ligaments to test how elastin content and distribution affect the mechanical response of the ligaments.

## Methods

### Multiphoton imaging of porcine knee collateral ligaments

**Fig. 1:**
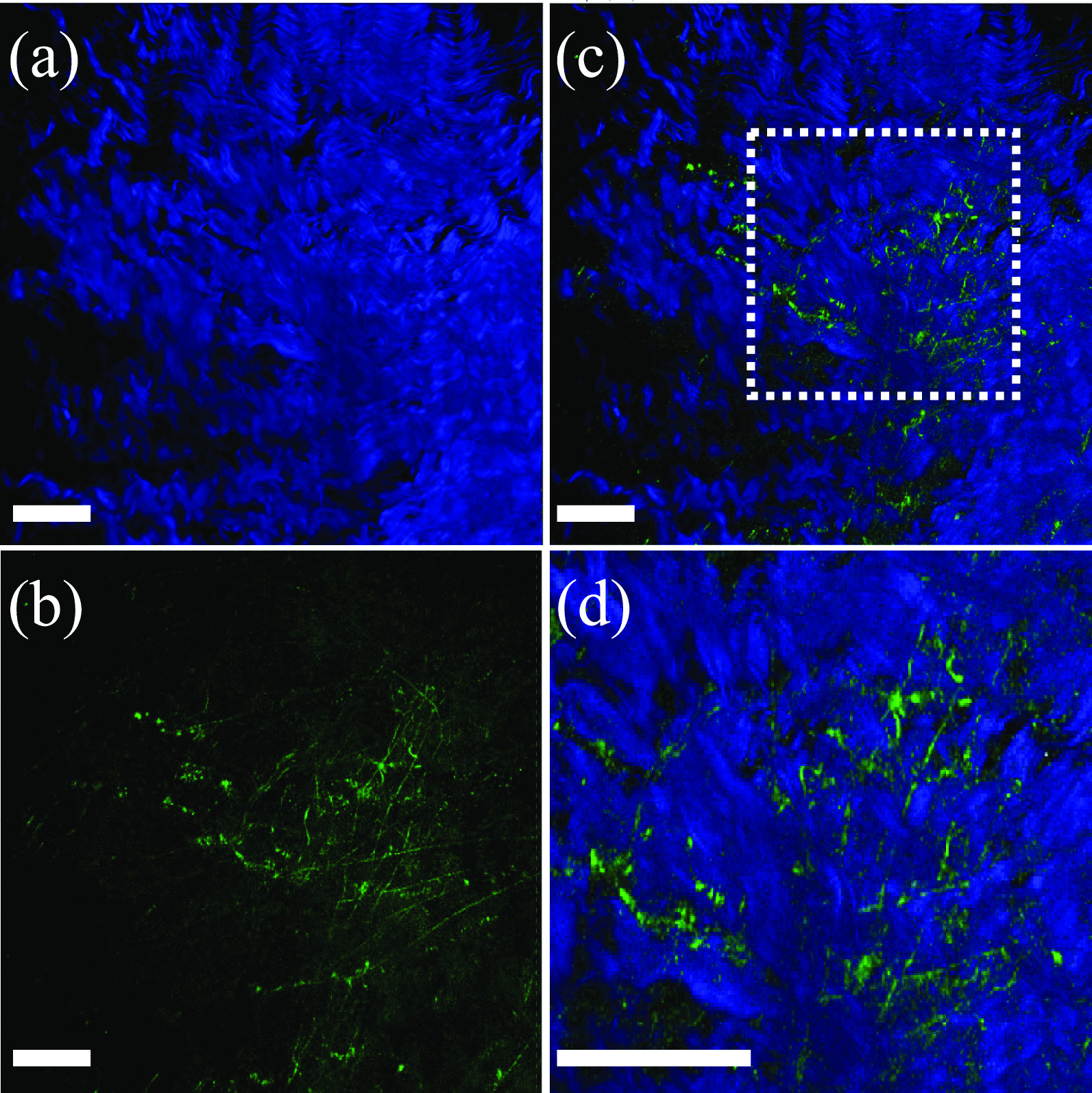
Multiphoton microscopic images of the porcine knee collateral ligament. **a** Second harmonic generation (SHG) image of collagen acquired through a DAPI filter (460 nm) and displayed with blue pseudocolour. **b** Autofluorescence image of elastin acquired through a GFP filter (525 nm) and displayed with green pseudocolour. **c** Merged image of SHG (blue; collagen) and autofluorescence (green; elastin) images. **d** Enlarged view of the area enclosed by the white square in panel (**c**). Scale bar in each panel indicates 500 µm

All animal experiments were approved by the Institutional Animal Care and Use Committee (approval number: T2021-38) and were carried out according to the Guidelines for Care and Use of Laboratory Animals of Tokushima University. To reduce the number of experimental animals used, hind legs, including the knee joints of slaughtered pigs, were purchased from a meat processing company (Tokyo Shibaura Zouki, Tokyo, Japan), and collateral ligaments were excised and used in the experiments. For multiphoton microscopy, an A1R-MP upright microscope system (Nikon Inc., Tokyo, Japan) was used and controlled by the NIS-Element ver.5.02. software (Nikon Inc.). The samples were excited with an ultrashort pulsed laser (Chameleon Laser, Coherent, Santa Clara, CA, USA; wavelength: 810 nm, pulse duration: 10 fs; repetition rate: 80 MHz) through a 25× water immersion objective lens (CFI75 Water Dipping Series, Nikon Inc.; magnification: 25× ; numerical aperture: 1.1; working distance: 2 mm), and second harmonic generation (SHG) light of collagen and autofluorescence of elastin were acquired through DAPI (central wavelength: 460 nm, bandwidth: 80 nm) and GFP (central wavelength: 525 nm, bandwidth: 40 nm) bandpass filters, respectively [13, 20]. Images were acquired with a resolution of 1024 × 1024 pixels, acquisition rate of 0.5 frames/s, and no accumulation. As shown in Fig. 1, we observed interstitial elastin deposition between the collagen fibres, which is consistent with previous reports [13]. The obtained images were analysed using Fiji ImageJ version 1.53f51 to measure the number of pixels of both blue and green colour [21]. The elastin content was estimated from the ratio of the green pixels to the total number of blue and green pixels. The elastin content of the porcine knee collateral ligament was 16.4%.

### Simulation model

**Fig. 2:**
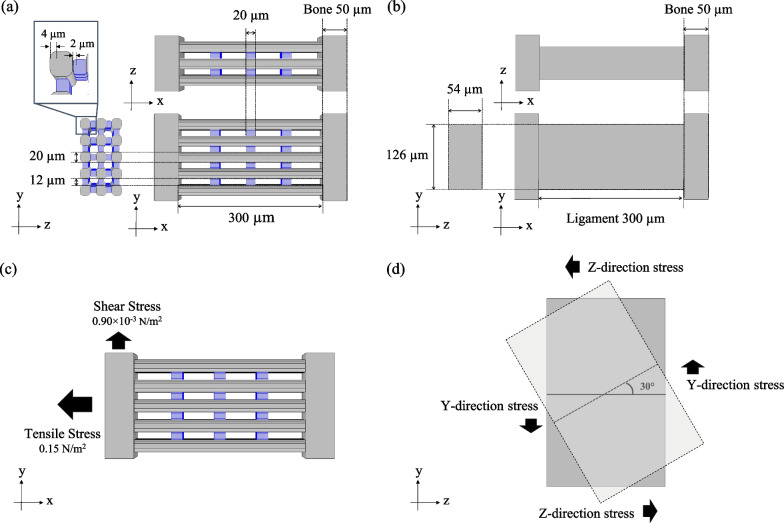
Mathematical simulation models. *X*-axis is set in the direction of tensile stress applied to the model, *Y*-axis is the direction of shear stress applied to the model, and *Z*-axis is orthogonal to both *X*- and *Y*-axes. **a** Structural diagram of a fibre model consisting of collagen and elastin (placed between collagen fibres, shown in blue). **b** Structural diagram of a sheet model with the same volume as the fibre model shown in (**a**). **c** Directions and strength of tensile and shear stresses applied to the model. **d** The directions of the stresses applied to rotate the bone by 30°

Based on multiphoton microscopic images, a simple mathematical model of the existing parallel-to-collagen fibres was designed using the mathematical simulation software COMSOL Multiphysics version 6.1 (COMSOL AB, Stockholm, Sweden). We also referred to the scanning electron microscopy images of ligament cross sections in previous literature [22]. We constructed a model in which the ligament was composed of multiple collagen fibres (fibre model). A single collagen fibre was configured as a square prism with a width and height of 20 µm, a length of 300 µm, and rounded corners on the long axis side. A single elastin fibre was also configured as a square prism with a width and height of 12 µm, and a length of 20 µm, and rounded corners on the long axis side. The content in the model was varied from 0 to 33.5% to evaluate the influences of elastin content on the mechanical properties of the ligament. As shown in Fig. 2a, three rows × five columns of collagen fibres were placed at equal intervals, with both ends attached to the bones. Elastin was placed at equal intervals between the collagen fibres, and both fibres were attached in parallel. A model in which the ligament was considered as a single sheet (sheet model) was also constructed with the same volume as the fibre model, containing 14.4% elastin, measuring 62 µm in width, 128 µm in height, and 300 µm in length (Fig. 2b). Table 1 shows the mechanical properties of collagen, elastin, and bone based on previous studies [13, 23, 24]. In this study, the boundary condition between collagen and elastin was set as complete fixation to simplify the model and clarify the effect of elastin on the mechanical responses of the ligament.

**Table 1:** Mechanical properties of each component in mathematical models

	Ligament			Bone
	Fibre model		Sheet model	
	Collagen	Elastin		
Young’s modulus [Pa]	8.9 × 10^6^	4.0 × 10^6^	12.7 × 10^6^	17.0 × 10^6^
Poisson’s ratio	0.48	0.48	0.48	0.30

The Young’s modulus of the collagen and elastin components were obtained from or calculated based on Henninger et al. [13]. Poisson’s ratios of the collagen and elastin components were obtained from or calculated based on Swedberg et al. [23]. The mechanical parameters of the bone were obtained from or calculated based on Lai et al. [24]

One bone in each model was fixed in a static-balance condition, and tensile, shear, or rotational stress was applied to the other bone. Based on previous experimental data [13], a stress of 0.15 N/m^2^ in the tensile direction or 0.90 × 10^–3^ N/m^2^ in the shear direction was applied, as shown in Fig. 2c, to compare the magnitude of stress applied to collagen and elastin and the extensibility of each component. Based on previous simulation data showing that the tibia rotated by approximately 30° when the knee was bent [17], the stresses were applied so that the bone on the non-fixed side rotated by 30°, as shown in Fig. 2d. The stress in *Y* and *Z* directions required for the bone to rotate 30° was measured, while elastin content was varied from 0 to 33.5%.

## Results

### Comparison between sheet and fibre models under stress

**Fig. 3:**
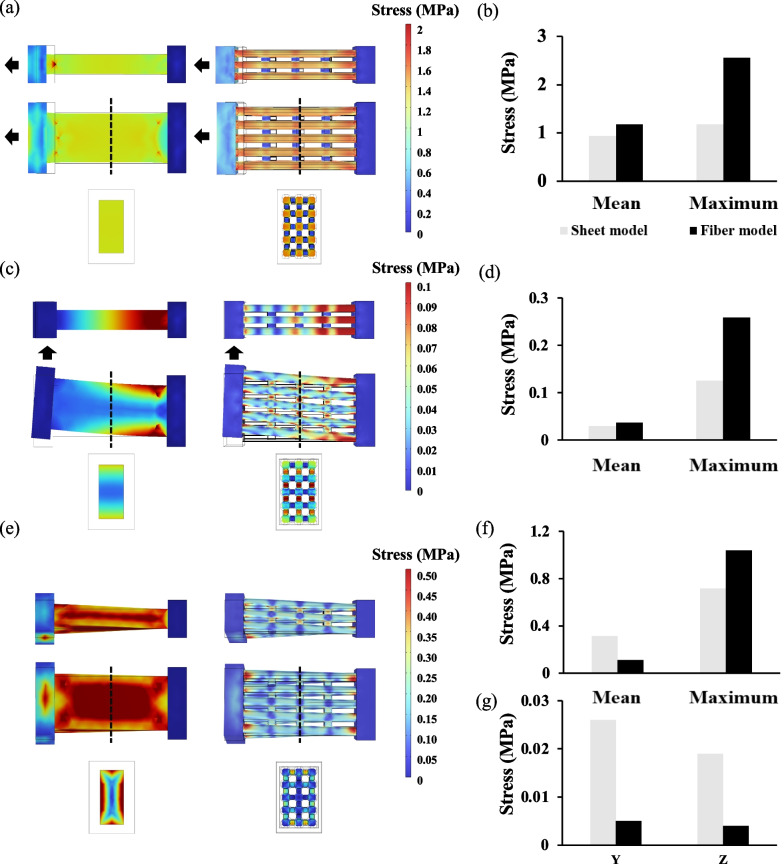
Differences in stress distribution between sheet and fibre models with 14.4% elastin. **a** Stress distribution in the sheet model (left) and the fibre model (right) under tensile stress. The *XZ*-plane (top), *XY*-plane (medium), and cross section in the *YZ*-plane at the dotted line in the centre of the model (bottom) are shown, respectively. The black arrow indicates the direction in which tensile stress was applied. **b** Summarised data of average and maximum stress in the sheet model (white boxes) and the fibre model (black boxes) under tensile stress. **c** Stress distribution in the sheet model (left) and the fibre model (right) under shear stress. The *XZ*-plane (top), *XY*-plane (medium), and cross section in the *YZ*-plane at the dotted line in the centre of the model (bottom) are shown, respectively. The black arrow indicates the direction in which shear stress was applied. **d** Summarised data of average and maximum stress in the sheet model (white boxes) and the fibre model (black boxes) under shear stress. **e** Stress distribution in the sheet model (left) and the fibre model (right) when the bone was rotated by 30°. The *XZ*-plane (top), *XY*-plane (medium), and cross section in the *YZ*-plane at the dotted line in the centre of the model (bottom) are shown, respectively. **f** Summarised data of average and maximum stress in the sheet model (white boxes) and the fibre model (black boxes) when the bone was rotated by 30°. **g** Stress in *Y* and *Z* directions required to rotate the bone by 30° in the sheet model (white boxes) and the fibre models (black boxes)

Figure 3a shows the stress distributions in the sheet and fibre models when tensile stress was applied. In the sheet model, the stress was uniformly distributed over the entire ligament. In contrast, in the fibre model with 14.4% elastin, a stress concentration was observed at the junction between the collagen fibre and bone. The stress on each collagen fibre was greater than that in the sheet model; however, the stress on the collagen fibres was reduced in the elastin-connected area. The data summarised in Fig. 3b show that the maximum stress on the collagen fibres was larger in the fibre model than in the sheet model. Under shear stress, the sheet model revealed strong stress applied to the flexion–extension area, whereas the fibre model showed stress applied to the entire collagen fibre (Fig. 3c). However, the maximum stress applied to the collagen fibres was approximately twice as high in the fibre model than in the sheet model under tensile stress (Fig. 3d). In addition, very strong stress was applied to elastin under shear stress, unlike tensile stress, where very little stress was applied to elastin. Figure 3e shows the stress distribution when the bone is rotated by 30°. Strong stress was distributed on the surface of the ligament in the sheet model, whereas stress was distributed on the elastin placed outside the ligament in the fibre model. Unlike the tensile and shear stresses, the average stress applied to the collagen fibres under rotational stress was smaller in the fibre model than in the sheet model, and the maximum stress in the fibre model was approximately 1.3 times that in the sheet model (Fig. 3f). Figure 3g shows the stress required to rotate the bone by 30°. The sheet model required approximately five times the stress to rotate the bone by 30° compared to the fibre model, suggesting that the rigidity of the ligament was significantly higher in the sheet model.

We also constructed a fibre model with 10.1% elastin and compared it with a sheet model of the same volume (Additional file 1: Fig. S1). Although the difference in the values was smaller than when the elastin content was 14.4%, a similar trend was observed in the stress distribution. The stress was uniformly distributed in the sheet model, whereas in the fibre model, the stress was stronger at the boundaries between the collagen fibres, bone, and elastin. In addition, elastin was also subjected to high stress when shear and rotational stresses were applied.

### Variation in ligament stress in the absence and presence of elastin

**Fig. 4:**
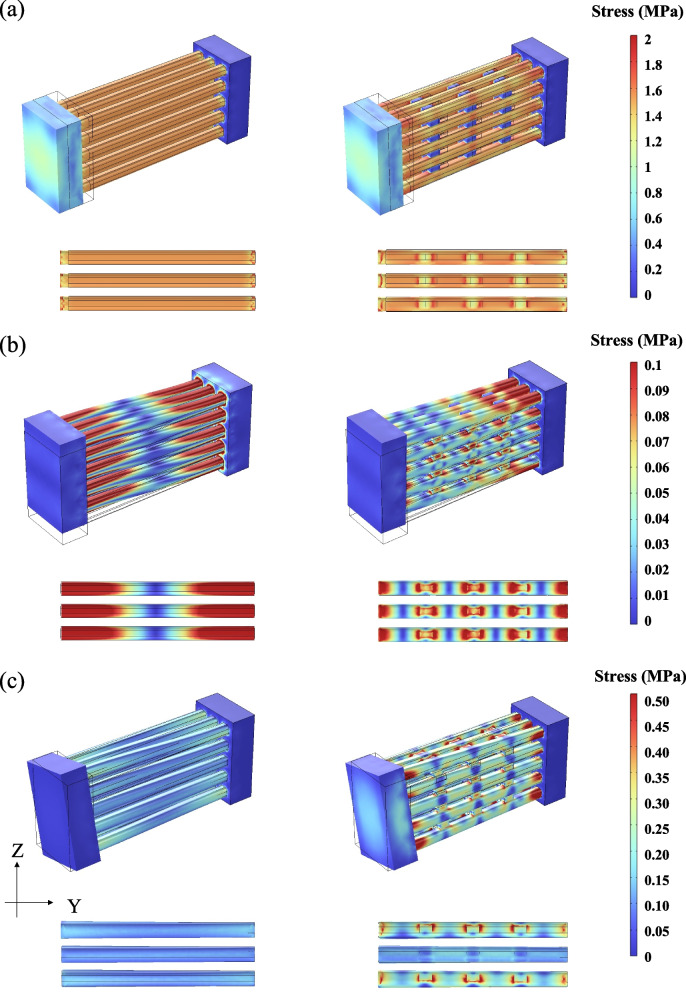
Difference in stress distribution in the fibre model in the absence and the presence of 14.4% elastin. **a** Differences in stress distribution between the models in the absence (left) and the presence (right) of elastin under tensile stress.** b** Differences in stress distribution between the models in the absence (left) and the presence (right) of elastin under shear stress. **c** Differences in stress distribution between the models in the absence (Left) and the presence (Right) of elastin when the bone was rotated by 30°. In each panel, the upper surface of the collagen fibres on the third row is shown at the bottom

Figure 4a shows the stress distribution when tensile stress was applied to the fibre model in the presence and absence of elastin. Stress was uniformly applied to collagen fibres in the absence of elastin (left panels). In contrast, in the presence of elastin, the stress on the collagen fibres was smaller, especially near the elastin-connected area, and the stress was concentrated at the collagen-elastin junction (right panels). Figure 4b shows the stress distribution when shear stress was applied to the fibre model in the presence and absence of elastin. In the absence of elastin, strong stress was applied mainly at the collagen-bone junction (left panels). In contrast, in the presence of elastin, stress was concentrated at the collagen-elastin junction (right panels), as was the case when tensile stress was applied. Figure 4c shows the stress distribution of the fibre model in the presence and absence of elastin when the bone was rotated by 30°. Although a simple comparison was difficult owing to the different stresses required to rotate the bone by 30°, the stress was stronger near the bone as well as the shear stress in the absence of elastin (left panels), whereas it was stronger on elastin and collagen in the presence of elastin (right panels). In addition, especially in the presence of elastin, stress concentration occurred at the junction of the collagen fibres and elastin, as well as when tensile and shear stresses were applied.

We also compared the stress distribution between the fibre models with an elastin content of 10.1% and those without elastin under various stresses (Additional file 1: Fig. S2). As in the case of 14.4% elastin content, the stress applied to the collagen was reduced at the elastin junction, and strong stress was observed to be applied to the elastin, especially when shear or rotational stress was applied.

### The influences of elastin content on stress and ligament displacement

**Fig. 5:**
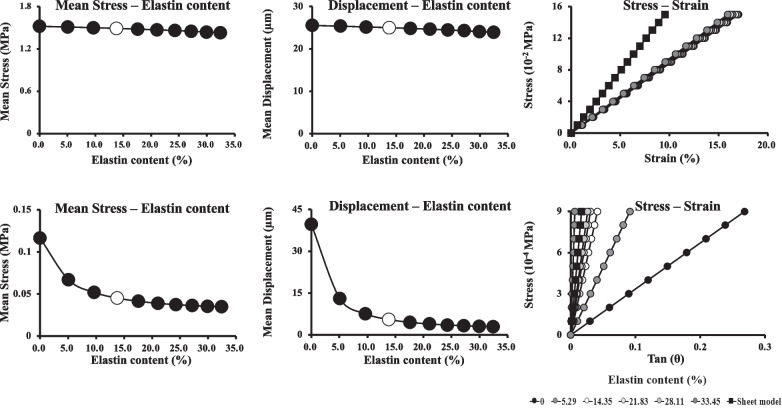
Relationship between stress and displacement of collagen by elastin content. **a** Changes in stress (left), displacement (middle), and stress–strain relationship (right) of collagen fibres under tensile stress when elastin content was varied from 0 to 33.5%. **b** Changes in stress (left), displacement (middle), and stress–strain relationship (right) of collagen fibres under shear stress when elastin content was varied from 0 to 33.5%. The open circles show the stress and displacement when the model was constructed with an elastin content of 18.3%, which was closest to the elastin content of 16.4% calculated from multiphoton microscopy images of the actual porcine knee collateral ligaments

Figure 5 shows how the mean stress, mean displacement of collagen, and stress–strain relationship changed with the elastin content. When tensile stress was applied, there was no significant difference in the stress–strain relationship as the elastin content varied from 0 to 33.5%, with only a slight decrease in stress applied to collagen by approximately 3% and collagen displacement by approximately 6% (Fig. 5a). However, when shear stress was applied, increasing the elastin content from 0 to 33.5% resulted in an exponential decrease in the stress and displacement of collagen by approximately 65% and 89%, respectively. Furthermore, the slope of the stress–strain relationship steepens with increasing the elastin content, suggesting that elastin significantly increases the rigidity of the ligament against shear stress. Another point was that the slope of the stress–strain relationship in the case of the sheet model was larger those that of the fibre models, and the difference was particularly pronounced when tensile stress was applied. It was suggested that the sheet model may overestimate the rigidity of the ligament compared to the fibre model. Table 2 lists the stresses required to rotate the bone by 30° in the *Y* and *Z* directions. As the elastin content increased from 0 to 33.5%, the required stresses increased linearly, indicating that elastin may contribute to the rigidity of the ligament against rotational and shear stresses. It should be noted that the stress required to rotate the bone by 30° in the sheet model was significantly greater than in the fibre model, indicating that the sheet model cannot accurately reflect the mechanical response of the ligament to rotational stresses.

**Table 2:** Changes in stress required to rotate bone by 30° upon varying the elastin content

Elastin content [%]	*Y* direction stress [MPa]	*Z* direction stress [MPa]
0	0.002	0.001
5.3	0.003	0.003
10.1	0.005	0.004
14.4	0.006	0.005
18.3	0.008	0.007
21.8	0.009	0.009
25.1	0.011	0.010
28.1	0.012	0.011
30.9	0.014	0.013
33.5	0.015	0.014
Sheet model	0.026	0.019

## Discussion

In this study, we developed an original and simple mathematical simulation model of the collateral ligament based on two-photon images and previous studies. While conventional simulations often consider the ligament as a single sheet or bundle of collagen fibres [17, 19], our model is unique in that elastin, which has its own mechanical properties, is placed between the collagen fibres. We compared this unique fibre model with a conventional sheet-like model and found that the distribution and magnitude of stress on the ligament, as well as the displacement of collagen fibres during tensile, shear, and rotational stresses, differed significantly between the two models. In addition, we found that even in the same fibre model, the stress applied to the collagen fibres during tensile and shear stresses varied with the elastin content and that the stress required to rotate the bones placed at both ends of the ligament at the same angle increased with increasing the elastin content. These results suggest that elastin contributes to the maintenance of tissue rigidity when ligaments are subjected to shear and rotational stresses.

Previous studies using mathematical models that considered the ligament as a single sheet evaluated the stresses on the ligament macroscopically by inputting the mechanical properties measured on the actual excised ligament into the model [18]. In these cases, the mechanical properties of the ligament could be the average of those of collagen, elastin, and other components of the ligament. The sheet model we constructed for comparison with our original fibre model also inputted the mechanical properties of the ligament based on previous literature.

Previous fibre models that focussed specifically on collagen fibres employed only information on the mechanical properties of collagen and excluded the mechanical properties of elastin and other components [17]. Our present simplified mathematical simulation model consists of ligaments with collagen bundles and elastin between them, which account for more than 90% of the dry weight of the ligament [25]. Collagen and elastin were individually input with their own mechanical properties. The results of the present study indicate that the sheet model could only reflect the average stress distribution, whereas the fibre model could reveal a more precise stress distribution and mechanical response. In addition, this study showed that the role of elastin differed depending on the mechanical load applied to the ligament. Interestingly, there was little difference in rigidity with increasing elastin content under tensile stress, whereas the rigidity increased significantly with increasing elastin content under shear and rotational stress. Such differences in the mechanical properties of ligaments due to differences in elastin might be recognised clinically or experimentally as differences in mechanical properties depending on the type and location of the ligament. These can be ligaments in joints that require rigidity to withstand high loads (e.g. human knees), ligaments in joints that require a wide range of motion (e.g. shoulders), or even the same ligament near its junction with the bone and near its centre [9]. These results suggest that collagen mainly maintains the rigidity of the ligament under tensile stress, and elastin is mainly responsible for the rigidity of the ligament under shear and rotational stress. Thereby, we provide novel insights into the mechanism of ligament rupture under different loading directions. Moreover, in a previous experiment involving rats with type-2 diabetes, a decrease in elastin levels was associated with a decrease in skin stiffness when bulging stress was applied [26]. Unlike ligaments and tendons, where collagen is oriented in one direction, the collagen orientation in the skin is random. The involvement of elastin in skin tissue stiffness is supported by our experimental results.

Generally, when a force is applied to an object, the stress is concentrated in the area where the shape of the object changes or in fixed parts of the object, causing its destruction. As a common finding in our model, strong stress was applied to the collagen-elastin junction. Thus, when a strong force is applied to the ligaments, damage or rupture begins at the collagen-elastin junction. In addition, as the elastin content increased, less stress was applied to the collagen fibres. This suggests that elastin reduces stress in collagen fibres to prevent their rupture. According to previous studies, type-I collagen accounts for 85–90% of normal human ligaments and skin, whereas type-III collagen, which is less rigid, temporarily increases to approximately 33% during the healing process for three weeks after injury, and type-III collagen is replaced by type-I collagen over the next two years [27, 28]. In contrast, in a rat Achilles-tendon injury model, the elastin component in the tendon increased twofold during the first month after tendon injury [29]. These findings suggest that the intercalation of elastin between collagen fibres prevents damage to mature type-I collagen, which takes longer to repair, by concentrating stress on the collagen-elastin junction, and that elastin, which increases to twice its normal level during injury, protects type-III collagen, which is less rigid. Furthermore, these results may elucidate the mechanism of ligament damage and rupture due to fatigue accumulation.

The present study indicates that elastin was more stressed than collagen during shear stress and rotation (Fig. 4), which may cause damage and rupture. When the elastin content was lowered by repeated stress and damage over a shorter period than recovery, rigidity decreased, as shown in Fig. 5. An elastin content below 5% is thought to cause a sudden drop in rigidity, leading to excessive stretching and tearing. Thus, elastin damage may cause a sudden decrease in rigidity when its content falls below a certain threshold, which can easily result in damage or rupture. Conventional sheet or fibre models without elastin did not provide information for predicting such molecular or microscopic events. The present fibre model with elastin, albeit simple, provides useful information for inferring events at the microscopic level. In the future, a more sophisticated model, including elastin, will be able to accurately predict mechanical responses such as deformation and fracture when force is applied to the ligaments.

### Limitations

Several limitations exist in the present study since the models have a very simplified structure and were constructed without some physical parameters. Our models were constructed to contain collagen fibres lined in a straight rectangular shape with gaps between them. Collagen fibre bundles have a wavy helical structure, and adjacent collagen fibres are in contact with each other [16]. When tensile stress is applied to a model with such a structure, there is a process of straightening of the wavy shape, thus forming a toe region where only the strain increases without an increase in stress. In addition, many biological samples are not normally elastic but viscoelastic, and experiments using actual ligaments and other materials have shown that they exhibit nonlinear mechanical responses [30]. Even when tensile stress is applied to these tissues, they are expected to show a linear mechanical response in accordance with the elasticity of the tissue when the wavy shape is linearized, and the force applied exceeds the viscosity of the tissue. Our model constructed the ligament as an elastic body but not a viscoelastic body, which was one of the major limitations of this study. Another limitation is that the model is isotropic. In the isotropic model, the shear modulus is calculated from Young's modulus and Poisson's ratio to determine the stiffness and elasticity of the entire sample. However, actual ligaments and soft tissues are anisotropic, and the shear modulus cannot be simply determined from Young's modulus and Poisson's ratio alone. Nevertheless, it is important to note that even in this very simple model, elastin was shown to play a different role in the linear mechanical response, depending on the elastin content and the stress applied to the ligament. In the future, it is expected that an anisotropic model will be constructed as a viscoelastic body with a shape more similar to that of the actual ligament, allowing for very precise simulations.

The effect of crosslinkers on the mechanical response of ligaments should also be considered. Crosslinkers, such as laminin and proteoglycans, may be involved in the binding between collagen fibres or between collagen and elastin, which can strongly affect the strength of adhesion and friction between fibres and have a significant impact on the mechanical response of the entire ligament. The adhesion and friction due to crosslinkers between fibres can be simulated using parameters of the boundary conditions in simulation studies. However, it is difficult to obtain specific numerical data on the adhesion and friction strengths dictated by the crosslinker from tensile tests of ligament tissues. Additionally, the distributions of collagen and elastin within the ligament can be easily determined by SHG and autofluorescence microscopy, respectively, whereas these adhesion molecules must be observed by immunostaining. Previous studies confirmed that these components are widely but sparsely distributed throughout ligaments [9]. For these reasons, we set boundary conditions between the fibres for complete fixation, which is one of the major limitations of the present study. In the future, it is expected that the adhesion strength and friction between collagen and elastin will be varied in several ways, and that numerical data on crosslinker adhesion and friction will be estimated from a model that more closely resembles the mechanical properties of actual ligaments.

Even with this simple model, we found that the stress–strain relationship varied with the elastin content and distribution, as well as the type of stress applied to the ligament. Such information would be useful for the generation of novel biomaterials, such as, for example, the creation of artificial ligaments [31]. In addition, the inclusion of elastin in precise simulations may lead to the elucidation of ligament damage and rupture mechanisms and the prevention of ligament injuries.

## Conclusions

A simple fibre model that includes the mechanical properties of elastin can provide a more precise stress distribution and mechanical response of the ligament. A simulation study revealed that elastin was responsible for ligament rigidity under shear and rotational stresses.

## Supplementary Information

**Additional file 1:** The simulation model construction and the results of the mechanical response in each model when the elastin content was set to 10.1% are summarized. **Fig. S1.** Differences in stress distribution between sheet and fibre models with 10.1% elastin. **a** Stress distribution in the sheet model (left) and the fibre model (right) under tensile stress. *XZ*-plane (top), *XY*-plane (medium), and cross-section in the *YZ*-plane at the dotted line in the centre of the model (bottom) are shown, respectively. Black arrow indicates the direction in which tensile stress was applied. **b** Summarized data of average and maximum stress in the sheet model (white boxes) and the fibre model (black boxes) under tensile stress. **c** Stress distribution in the sheet model (left) and the fibre model (right) under shear stress. *XZ*-plane (top), *XY*-plane (medium), and cross-section in the *YZ*-plane at the dotted line in the center of the model (bottom) are shown, respectively. Black arrow indicates the direction in which shear stress was applied. **d** Summarized data of average and maximum stress in the sheet model (white boxes) and the fibre model (black boxes) under shear stress. **e** Stress distribution in the sheet model (left) and the fibre model (right) when the bone was rotated by 30°. *XZ*-plane (top), *XY*-plane (medium), and cross-section in the *YZ*-plane at the dotted line in the centre of the model (bottom) are shown, respectively. **f** Summarized data of average and maximum stress in the sheet model (white boxes) and the fibre model (black boxes) when the bone was rotated by 30°. **g** Stress in *Y* and *Z* directions required to rotate the bone by 30° in the sheet model (white boxes) and the fibre models (black boxes). **Fig. S2.** Difference in stress distribution in the fibre model in the absence and the presence of 10.1% elastin. **a** Difference in stress distribution between the models in the absence (Left) and the presence (Right) of elastin under tensile stress. **b** Differences in stress distribution between the models in the absence (Left) and the presence (Right) of elastin under shear stress. **c** Differences in stress distribution between the models in the absence (Left) and the presence (Right) of elastin when the bone was rotated by 30°. In each panel, the upper surface of the collagen fibers on the third raw are shown at the bottom.

## Data Availability

The datasets regarding this study are available from the authors upon request.

## Abbreviations

ECM: Extracellular matrix
SHG: Second harmonic generation
