# Supplementary material for: Elastin is responsible for the rigidity of the ligament under shear and rotational stress: a mathematical simulation study
Source: J Orthop Surg Res. 2023 Apr 19;18:310. doi: 10.1186/s13018-023-03794-6 (PMC10114388; doi:10.1186/s13018-023-03794-6)
Supplement: Supplementary file 1 — Additional file 1: The simulation model construction and the results of the mechanical response in each model when the elastin content was set to 10.1% are summarized. Fig. S1. Differences in stress distribution between sheet and fibre models with 10.1% elastin. a Stress distribution in the sheet model (left) and the fibre model (right) under tensile stress. XZ-plane (top), XY-plane (medium), and cross-section in the YZ-plane at the dotted line in the centre of the model (bottom) are shown, respectively. Black arrow indicates the direction in which tensile stress was applied. b Summarized data of average and maximum stress in the sheet model (white boxes) and the fibre model (black boxes) under tensile stress. c Stress distribution in the sheet model (left) and the fibre model (right) under shear stress. XZ-plane (top), XY-plane (medium), and cross-section in the YZ-plane at the dotted line in the center of the model (bottom) are shown, respectively. Black arrow indicates the direction in which shear stress was applied. d Summarized data of average and maximum stress in the sheet model (white boxes) and the fibre model (black boxes) under shear stress. e Stress distribution in the sheet model (left) and the fibre model (right) when the bone was rotated by 30°. XZ-plane (top), XY-plane (medium), and cross-section in the YZ-plane at the dotted line in the centre of the model (bottom) are shown, respectively. f Summarized data of average and maximum stress in the sheet model (white boxes) and the fibre model (black boxes) when the bone was rotated by 30°. g Stress in Y and Z directions required to rotate the bone by 30° in the sheet model (white boxes) and the fibre models (black boxes). Fig. S2. Difference in stress distribution in the fibre model in the absence and the presence of 10.1% elastin. a Difference in stress distribution between the models in the absence (Left) and the presence (Right) of elastin under tensile stress. b Differences in stress distribution be [file 13018_2023_3794_MOESM1_ESM.docx]

***Supplementary data for***

**Elastin is responsible for the rigidity of the ligament under shear and rotational stress: A mathematical simulation study**

**Yuki Naya, Hiroki Takanari**

**Simulation model**

We have validated the values and distribution of stresses in the ligament model at 14.4% elastin content in the text, and we have similarly validated the model at 10.1% elastin content. The specific methods for model construction are the same as in the text. Briefly, a simple mathematical model of existing parallel to collagen fibers was designed using a mathematical simulation software COMSOL Multiphysics ver.6.1 (COMSOL AB, Stockholm, Sweden) based on multiphoton microscopy and the previous literature [22]. We constructed a model in which the ligament was composed of multiple collagen fibers (fiber model). A single collagen fiber was set as a square prism with a width and height of 20 µm and a length of 300 µm, with rounded corners on the long axis side. A single elastin fiber was also set as a square prism with a width and height of 12 µm and with a length of 20 µm, with rounded corners on the long axis side. Three rows by five columns collagen fibers were placed at equal intervals with both ends attached to bones. Elastin was placed at equal intervals in between collagen fibers, so that the ratio of elastin volume to the total volume of collagen and elastin was 10.1%. A model in which the ligament was considered as a single sheet (a sheet model) was also constructed with the same volume as the fiber model containing 10.1% elastin, measuring 52 µm in width, 124 µm in height, and 300 µm in length. The mechanical properties of collagen, elastin, and bone were calculated and set individually based on the previous literatures [13, 23, 24]. The boundary condition between collagen and elastin was set as complete fixation to simplify the model.

**Results**

***Comparison of sheet model and fiber model under stress***

Figure S1a shows stress distribution in a sheet model and a fiber model when tensile stress was applied. In the sheet model, the stress was uniformly distributed over the entire ligament. On the other hand, in the fiber model with 10.1% of elastin, stress concentration was found at the junction between the collagen fiber and the bone. The stresses on each collagen fiber were larger than those in the sheet model, however, the stress on the collagen fiber was reduced at the elastin connected area. Summarized data in Figure S1b shows that the maximum stress on collagen fiber was apparently larger in the fiber model than in the sheet model. Under shear stress, the sheet model revealed strong stress applied to the flexion-extension area, whereas the fiber model showed stress applied to the entire collagen fiber (Fig. S1c). However, the maximum stress applied to the collagen fiber was about twice as high in the fiber model as in the sheet model, as was under the tensile stress (Fig. S1d). In addition, very strong stress was applied to elastin under shear stress unlike tensile stress, where very little stress was applied to the elastin. Figure S1e shows the stress distribution when the bone was rotated 30˚. Strong stress was distributed on the surface of the ligament in the sheet model, whereas the stress was distributed on the elastin placed outside of the ligament in the fiber model. Unlike tensile and shear stress, the average stress applied to the collagen fibers under rotational stress was smaller in the fiber model than in the sheet model, and the maximum stress in the fiber model was approximately 1.3 times that in the sheet model (Fig. S1f). Figure S3g shows the stress required to rotate the bone by 30˚. The sheet model required approximately five times the stress to rotate the bone by 30˚ when compared to the fiber model, suggesting that the rigidity of the ligament would be significantly higher in the sheet model.

**Figure S1.** Differences in stress distribution between sheet and fiber models with 10.1% elastin. **(*a*)** Stress distribution in the sheet model (*left*) and the fiber model (*right*) under tensile stress. XZ-plane (*top*), XY-plane (*medium*), and cross-section in the YZ-plane at the dotted line in the center of the model (*bottom*) are shown, respectively. Black arrow indicates the direction in which tensile stress was applied. **(*b*)** Summarized data of average and maximum stress in the sheet model (white boxes) and the fiber model (black boxes) under tensile stress. **(*c*)** Stress distribution in the sheet model (*left*) and the fiber model (*right*) under shear stress. XZ-plane (*top*), XY-plane (*medium*), and cross-section in the YZ-plane at the dotted line in the center of the model (*bottom*) are shown, respectively. Black arrow indicates the direction in which shear stress was applied. **(*d*)** Summarized data of average and maximum stress in the sheet model (white boxes) and the fiber model (black boxes) under shear stress. ***(e*)** Stress distribution in the sheet model (*left*) and the fiber model (*right*) when the bone was rotated by 30˚. XZ-plane (*top*), XY-plane (*medium*), and cross-section in the YZ-plane at the dotted line in the center of the model (*bottom*) are shown, respectively. **(*f*)** Summarized data of average and maximum stress in the sheet model (white boxes) and the fiber model (black boxes) when the bone was rotated by 30˚. **(*g*)** Stress in Y and Z directions required to rotate the bone by 30˚ in the sheet model (white boxes) and the fiber models (black boxes).

***Variation in stress on ligament in the absence and presence of elastin***

Figure S2a shows the stress distribution when tensile stress was applied to a fiber model in the presence and absence of elastin. The stress was uniformly applied to the collagen fibers in the absence of elastin (*left panels*). On the other hand, in the presence of elastin, the stress on collagen fibers was smaller especially near the elastin-connected area, and the stress was concentrated at the collagen-elastin junction instead (*right panels*). Figure S2b shows the stress distribution when shear stress was applied to the fiber model in the presence and absence of elastin. In the absence of elastin, strong stress was applied mainly at the collagen-bone junction (*left panels*). On the other hand, in the presence of elastin, stress was concentrated at the collagen-elastin junction, as was the case when tensile stress was applied (*right panels*). Figure S2c shows the stress distribution for the fiber model in the presence and absence of elastin when the bone was rotated by 30˚. Although a simple comparison was difficult due to the different stresses required to rotate the bone 30˚, roughly, the stress was stronger near the bone as well as the shear stress in the absence of elastin, while the stress was stronger on the elastin as well as the collagen in the presence of elastin. In addition, especially in the presence of elastin, stress concentration occurred at the junction of collagen fibers and elastin as well as when tensile and shear stress was applied.

**Figure S2.** Difference in stress distribution in the fiber model in the absence and the presence of 10.1% elastin. **(*a*)** Difference in stress distribution between the models in the absence (*Left*) and the presence (*Right*) of elastin under tensile stress. **(*b*)** Differences in stress distribution between the models in the absence (*Left*) and the presence (*Right*) of elastin under shear stress. **(*c*)** Differences in stress distribution between the models in the absence (*Left*) and the presence (*Right*) of elastin when the bone was rotated by 30˚. In each panel, the upper surface of the collagen fibers on the third raw are shown at the bottom.
